# Cesarean delivery technique among HIV positive women with sub-optimal antenatal care uptake at the Douala General Hospital, Cameroon: case series report

**DOI:** 10.1186/s13104-017-2639-0

**Published:** 2017-07-26

**Authors:** Thomas Obinchemti Egbe, Charlotte Nguefack Tchente, George-Fulbert Mangala Nkwele, Jacques Ernest Nyemb, Esther Mathio Barla, Eugene Belley-Priso

**Affiliations:** 1 Faculty of Health Sciences, University of Buea, P.O. Box 63, Buea, Cameroon; 2 Douala General Hospital, P.O. Box 4856, Douala, Cameroon; 3 Faculty of Medicine and Pharmaceutical Sciences, University of Douala, Douala, Cameroon; 4 Faculty of Medicine and Biomedical Sciences, University of Yaounde 1, Yaounde, Cameroon

**Keywords:** Cesarean section, Vaginal delivery, Human immunodeficiency virus, Antenatal care visits, Viral load, CD4 count

## Abstract

**Background:**

The human immunodeficiency virus (HIV) pandemic is a serious public health problem worldwide, especially in low-income countries of sub-Saharan Africa (SSA). The prevention of mother to child transmission of HIV (PMTCT) is a major concern to those countries. Cesarean section has been described in the literature to be effective in the prevention of mother to child transmission (MTCT).

**Case series presentation:**

We present a series of seven cases of HIV positive pregnant women with sub-optimal antenatal care up-take who delivered by cesarean section at the Department of Obstetrics and Gynecology of the Douala General Hospital. During the cesarean section the fetal head was delivered through the uterine incision without rupture of amniotic membranes. The amniotic membranes were ruptured after delivery of the fetal head, and then the rest of the body was delivered.

**Conclusions:**

Most of the study participants had multiple risk factors for preterm labour. When a good cesarean section technique is used in women with high viral load and low CD4 counts, risk of MTCT HIV are greatly reduced even in low-income countries.

## Background

Cesarean section is one of the most commonly performed operations in women in both affluent and low-income countries and accounts for up to 60% of deliveries in some countries [1]. Global estimates indicate a cesarean section rate of 15% worldwide, ranging from 3.5% in Africa to 29.2% in Latin America and the Caribbean [2]. The World Health Organization (WHO) and the Pan American Health Organization suggest the ideal cesarean section rate for a country is 5–15% [3].

In the developing world, access to cesarean section is an indication of effective emergency obstetric care [3].

The number of people living with HIV has never been higher; UNAIDS estimates the global figure to be 34.2 million people. The human immunodeficiency virus (HIV) infection is now considered a chronic disease in the developing world, at a time when life expectancy is approaching that of the economically developed countries [4, 5]. Without antiretroviral treatment, estimated median time from seroconversion to death is approximately 10 years. With antiretroviral treatment, individuals with recently acquired HIV infection have nearly the same life expectancy as HIV-negative persons, provided they are diagnosed early, have extensive access to HIV care and treatment and maintain good adherence. Many HIV-positive women choose to pursue pregnancy [4, 5]. This has not been the case in many low-income countries in sub-Saharan Africa. Pooled data has shown that the HIV prevalence in sub-Saharan Africa declined among pregnant women from 6.5% [95% CI 5.3–7.9] to 5.3% [95% CI 4.2–6.6] [6].

Management of the HIV-positive pregnant patient should focus on both decreasing the risk of MTCT and minimizing maternal and neonatal complications. Where it is not treated, MTCT is about 30–40% in most low-income countries [7], and increases during labour and delivery by about 10–20% without intervention [7]. The risk of MTCT was significantly associated with a low level of CD4+ cells (<29%), a high level of CD8+ cells (>50%), a positive HIV-1 culture at delivery, prematurity, low birth weight, longer duration of ruptured membranes, premature rupture of membranes (that is, before the onset of labor), premature rupture of membranes in a preterm delivery (that is, before 37 weeks’ gestation), use of hard drugs during pregnancy, the presence of chorioamnionitis, and maternal age of at least 30 years [7–9].

In Cameroon, the prevalence of HIV was estimated at 4.3% in the general population; a serosurveillance survey among pregnant women showed an HIV prevalence of 7.6% in 2010 [10]. In 2011, Cameroon opted for the WHO Option B + regimen for PMTCT prophylaxis. Continuing access of pregnant women living with HIV to prenatal HIV services and increasing access to HIV treatment for eligible children and pregnant women will reduce maternal and child mortality [11]. Reports in Cameroon show that in 2010, PMTCT services were available to 99.4% health districts, that 2067 health facilities out of 3214 offered antenatal care and PMTCT services, and that only 38.26% of 970,306 pregnant women received ANC from health facilities providing ANC and PMTCT services while 30.1% were screened for HIV. The PMTCT programme coverage of HIV positive women was 23.47% [10, 11].

In Cameroon, HIV positive mothers are recommended exclusive breastfeeding and compliance with ARV treatment. They are also to avoid breast engorgement, nipple crackles and lactation mastitis; factors that could increase the risk of MTCT [7, 12].

The American College of Obstetricians and Gynecologists (ACOG) recommends that elective cesarean section (cesarean section before labor or rupture of membranes (ROMs)) be performed for delivery when viral load is detectable or greater than 1000 copies/ml as there is a 12-fold risk of MTCT [13]. This recommendation is based on several studies that show that the combination of intra-partum zidovudine (ZDV) and elective cesarean section significantly decreased vertical transmission compared to other delivery modes. With the addition of highly active antiretroviral therapy (HAART), the risk of vertical transmission has continued to decrease [5].

Antiretroviral therapy, which not only improves the health and well being of people living with HIV but also stops further HIV transmission, was available for 6.65 million people in low and middle-income countries. This number accounts for 47% of the 14.2 million people eligible [14]. Adherence rates of about 74% have been reported [15]. In the developing world, access to cesarean section is an indication of effective emergency obstetric care [3]. Given that postpartum morbidity from cesarean section is potentially higher in HIV-positive women, especially in low-income countries [16], achieving a vaginal delivery in this population of women is beneficial.

In Cameroon, and thanks to the national PMTCT program, HIV-positive women usually deliver vaginally. Cesarean section has a protective effect among patients with high viral load and low CD4 count. This study aims at describing the cesarean section technique employed among HIV-positive women with sub-optimal ANC uptake at the Douala General Hospital, Cameroon.

## Case series presentation

### Case 1

PK, a 23-year-old primigravida at 34 weeks’ gestation dated by last menstrual period (LMP) (10/04/2009), admitted to our Department with uterine contractions every 7 min that has lasted 12 h. She reports that fetal movements are present, denies leakage of fluid, vaginal bleeding, headaches, visual changes and right upper quadrant pain. Antenatal care was in a primary care centre (one visit at 24 weeks gestation, uterine size corresponding with gestational age, and blood pressure 100/65 mmHg). Problem list includes history of Positive Human Immunodeficiency virus test. Patient admitted in latent phase of labor with a vaginal exam (VE) Cervical dilatation 2 cm, cervix 50% effaced and station-5 (2/50/-5)

Past obstetric history: negative.

#### Past gynecological history

Menarche at 14 years old, menstrual cycle 30 days, regular. Past history of pelvic inflammatory disease (PID) and had never done a Pap-smear nor taken contraception. She is blood group A Rhesus positive, declares no allergies and received one dose of sulfadoxine−pyrimethamine for malaria prevention. She took iron supplements occasionally after 24 weeks.

Social history: Denies history of alcohol, smoking, drug abuse and has had two sexual partners.

Family history: Parents are not married. Declares no history of hypertension or diabetes mellitus.

Review of system: Abundant vaginal discharge, no fever, no chills.

#### Physical examination

- General appearance: not alert but oriented.
- Vital signs: Temperature = 38.5 °C, BP 100/65 mmHg, Pulse 96 bpm.
- Head, eye, ear, nose, throat (HEENT): No scleral icterus, pale conjunctiva.
- Neck: normal; lungs: normal; heart: normal; breasts: symmetric; no masses.
- Abdomen: gravid, non-tender.
- Fundal height: 30 cm.
- Presentation: Vertex.
- Extremities: mild lower extremity edema, non-pitting.
- Pelvis: Adequate.
- Sterile speculum exam (SSE): membranes intact, abundant offensive vaginal discharge.
- Sterile vaginal exam (SVE): 2 cm/50%/-5 (dilatation, effacement, station).
- Ultrasound: Vertex presentation confirmed, posterior placenta, no fetal anomalies, AFI = 12.1
- Fetal monitor: Baseline FHR: 162, accelerations present, no decelerations, no variability. Tocography = 1 UC q 7 min.

#### Assessment

23-year-old G1P0 at 34 weeks gestational age (GA) presented with regular painful contractions.

1. Latent phase of labor.
2. HIV positive.
3. Vaginal discharge.
4. H/o PID.

#### Plan

1. Admit to labor and delivery room.
2. Nothing per os.
3. Dextrose 5% at 125 ml/h.
4. Lab: Full blood count (FBC), Thick and thin blood films for malaria, blood electrolytes, coagulation studies, vaginal smear + culture, positive repeat determine HIV test, CD4 count, Viral load (VL), Aspatate aminotransferase, alanine aminotransferase, blood urea nitrogen (BUN),
5. Antiretroviral therapy.
6. External fetal monitor (EFM).
7. Type and cross-match blood.
8. Prepare for cesarean delivery.

#### Results

Hemoglobin: 5.2 g/dl; hematocrit: 15.4%; platelet count: 100,000; CD4:194; VL 17,000 copies/ml; vaginal smear: Gardnerella vaginalis positive (Metronidazol).

She underwent a cesarean delivery of a live-born female who weighed 2350 g, 5 min Apgar score 6, blood loss estimated at 600 ml. She was transfused 2000 ml packed cells and hospital stay was 10 days. Mother received bromocriptine for breast milk suppression and baby received exclusive formula milk. HIV testing of baby at 18 months was negative.

### Case 2

MS, a 26-year-old primigravida at 34 weeks’ gestation dated by last menstrual period admitted to our Department with elevated temperature (39 °C). Unemployed, single primary school leaver. She reports that fetal movements are present, denies leakage of fluid or vaginal bleeding. She has not done any prior antenatal care (ANC) visits. Problem list includes history of Positive Human Immunodeficiency virus test. Patient admitted with a vaginal exam (VE) showing Cervix long, posterior and closed.

Past obstetric history: negative.

#### Past gynecological history

Menarche at 12 years old, menstrual cycle 27 days, regular. Had never done a Pap-smear nor taken contraception. She is blood group O Rhesus positive, declares no allergies and received no antimalarial prophylaxis. Declares she has been taking iron supplements throughout pregnancy (without medical prescription).

Social history: Denies history of alcohol, smoking, drug abuse and has had more than four sexual partners.

Family history: Parents are divorced. Declares no history of hypertension or diabetes mellitus.

Review of system: fever, headaches and generalized joint pain.

#### Physical examination

- General appearance: alert and oriented.
- Vital signs: Temperature = 39 °C, BP 120/80 mmHg, Pulse 82 bpm.
- HEENT: No scleral icterus, pale conjunctiva.
- Neck: normal; lungs: normal; heart: normal; breasts: symmetric, no masses.
- Abdomen: Gravid, non-tender.
- Fundal height: 28 cm.
- Presentation: indeterminate.
- Extremities: mild lower extremity edema, non-pitting.
- Pelvis: Adequate.
- Sterile speculum exam (SSE): membranes intact, no vaginal discharge.
- Sterile vaginal exam (SVE): cervix long posterior closed.
- Ultrasound: vertex presentation confirmed, fundal placenta, no fetal anomalies.
- Fetal monitor: Baseline FHR: 170, accelerations and decelerations present, including variability.

#### Assessment

26-year old G1P0 at 34 weeks GA presented with elevated temperature.

1. Pyrexia of unknown origin (PUO).
2. HIV positive.

#### Plan

3. Admit to labor and delivery room.
4. Nothing per os.
5. Dextrose 5% at 125 ml/h.
6. Lab: Full blood count (FBC), thick and thin films for malaria, Widal-Felix serology, blood electrolytes, coagulation studies, urinalysis + culture, CD4 count, Viral load (VL), Aspatate aminotransferase, alanine aminotransferase, blood urea nitrogen (BUN),
7. Antiretroviral therapy.
8. External fetal monitor (EFM).
9. Type and cross-match blood.
10. Prepare for cesarean delivery.

#### Results

Thick film: positive for malaria and was treated with quinine infusions. Hemoglobin: 6.3 g/dl, hematocrit: 19.1%, platelet count: 80,000, CD4: 198, VL 15,000 copies/ml. CD4 and viral load results came after the cesarean delivery.

She underwent a cesarean delivery of a live-born female who weighed 2000 g. The 5 min Apgar score was 6 and estimated blood loss was 650 ml. She was transfused 1500 ml packed cells and hospital stay was 7 days. Mother received bromocriptine for breast milk suppression and baby received exclusive formula milk. HIV testing of baby at 18 months was negative.

### Case 3

DA, a 21-year-old primigravida at 33 weeks’ gestation dated by last menstrual period was admitted to our department because of elevated temperature (39 °C), diarrhea and wasting. Unemployed, single primary school leaver. She reports that fetal movements are present, and denies leakage of fluid or vaginal bleeding. She has not done any prior prenatal visit. Problem list includes history of Positive Human Immunodeficiency virus test done in the labour room. Patient admitted with a vaginal exam (VE) showing Cervix long, posterior and closed.

Past obstetric history: negative.

#### Past gynecological history

Menarche at 13 years old, menstrual cycle 30 days, regular. Had never done a Pap smear nor taken contraception. She is blood group AB Rhesus positive, declares no allergies and received no antimalarial prophylaxis. Declares she has not been taking iron supplements throughout pregnancy.

Social history: Denies history of alcohol, smoking, drug abuse and has had more than four sexual partners.

Family history: Parents are married with no salaried job. Declares no history of hypertension or diabetes mellitus.

Review of system: fever, diarrhea, anorexia, weight loss and fatigue.

#### Physical examination

- General appearance: wasted and oriented.
- Vital signs: Temperature = 39 °C, BP 95/60 mmHg, Pulse 100 bpm.
- HEENT: No scleral icterus, pale conjunctiva with slightly sunken eyes.
- Neck: normal, Lungs; normal, Heart; normal, Breasts; Symmetric, no masses.
- Abdomen: Gravid, non-tender.
- Presentation: vertex.
- Extremities: no lower extremity edema.
- Pelvis: Adequate.
- Sterile speculum exam (SSE): membranes intact, no vaginal discharge.
- Sterile vaginal exam (SVE): cervix long posterior closed.
- Ultrasound: vertex presentation confirmed, posterior placenta, no fetal anomalies.
- Fetal monitor: Baseline FHR: 155, accelerations and decelerations were present, including variability.

#### Assessment

21-year old G1P0 at 33 weeks GA presented with elevated temperature, diarrhea and wasting.

1. Febrile enteritis with dehydration.
2. HIV positive.

#### Plan

3. Admit to labor and delivery room.
4. Nothing per os.
5. D5 NS 2500 ml/24 h.
6. Lab: Full blood count (FBC), thick and thin films for malaria, blood electrolytes, coagulation studies, stool + culture, Widal-Felix serology, CD4 count, Viral load (VL), Aspatate aminotransferase, alanine aminotransferase, blood urea nitrogen (BUN),
7. Antiretroviral therapy.
8. External fetal monitor (EFM).
9. Type and cross-match blood.
10. Prepare for cesarean delivery.

#### Results

Thick film: positive for malaria and was treated with quinine infusions. Hemoglobin: 6.4 g/dl, hematocrit: 19.3%, platelet count: 65,000, CD4: 150, VL 18,000 copies/ml. CD4 and viral load results came after the cesarean delivery.

She underwent a cesarean delivery of a live-born female who weighed 2000 g. The 5 min Apgar score was 5 and estimated blood loss was 800 ml. She was transfused 2000 ml packed cells. Hospital stay was 12 days. Mother received bromocriptine for breast milk suppression and baby received exclusive formula milk. HIV testing of the baby was negative at 18 months.

### Case 4

MO, a 24-year-old primigravida at 35 weeks’ gestation dated by last menstrual period admitted to our Department with uterine contractions q 5 min. Unemployed, single primary school leaver. She reports that fetal movements are present, and denies leakage of fluid or vaginal bleeding. She has not done any prior prenatal visit. Problem list includes Positive HIV test done in the labour room. Patient admitted with a vaginal exam (VE) showing Cervix dilated at 4 cm, 80% effaced and station-2.

Past Obstetric history: negative.

#### Past gynecological history

Menarche at 11 years old, menstrual cycle 28 days, regular. Had never done a Pap smear nor taken contraception. She is blood group B Rhesus positive, declares no allergies and received no antimalarial prophylaxis. Declares she has not been taking iron supplements throughout pregnancy.

Social history: Denies history of alcohol, smoking, drug abuse and has had more than four sexual partners.

Family history: Parents are married and father has a low-income salaried job. Declares no history of hypertension or diabetes mellitus.

Review of system: fever, diarrhea, anorexia, weight loss and fatigue.

#### Physical examination

- General appearance: wasted and oriented.
- Vital signs: Temperature = 37.4 °C, BP 130/80 mmHg, Pulse 75 bpm.
- HEENT: No scleral icterus, pale conjunctiva.
- Neck: normal; lungs: normal; heart: normal; breasts: symmetric, no masses.
- Abdomen: gravid, non-tender.
- Presentation: vertex.
- Extremities: no lower extremity edema.
- Pelvis: adequate.
- Sterile speculum exam (SSE): membranes intact, no vaginal discharge.
- Sterile vaginal exam (SVE): cervix long posterior closed.
- Ultrasound: vertex presentation confirmed, posterior placenta, no fetal anomalies.
- Fetal monitor: baseline FHR: 155, accelerations and decelerations were present, including variability.

#### Assessment

24-year old G1P0 at 35 weeks GA presented with uterine contractions q 5 min.

1. Preterm labor in early active phase.
2. HIV positive.

#### Plan

1. Admit to labor and delivery room.
2. Nothing per os.
3. D5 RL 125 ml/h.
4. Lab: Full blood count (FBC), thick and thin films for malaria, blood electrolytes, coagulation studies, CD4 count, Viral load (VL), aspatate aminotransferase, alanine aminotransferase, blood urea nitrogen (BUN),
5. Antiretroviral therapy.
6. External fetal monitor (EFM).
7. Type and cross-match blood.
8. Prepare for cesarean delivery.

#### Results

 Thick film: positive for malaria and was treated with quinine infusions. Hemoglobin: 5.0 g/dl, hematocrit: 14.9%, platelet count: 75,000, CD4: 143, VL 20,000 copies/ml. CD4 and viral results were received after the cesarean delivery.

She underwent a cesarean delivery of a live-born male. The 5 min Apgar score was 6 and estimated blood loss was 800 ml. She was transfused 2000 ml packed cells. Hospital stay was 6 days. Mother received bromocriptine for breast milk suppression and baby received exclusive formula milk. HIV testing of the baby at 18 months was negative.

### Case 5

HB, a 20½-year-old primigravida at 33 weeks’ gestation dated by last menstrual period admitted to our Department with uterine contractions q 10 min. Unemployed, single primary school leaver. She reports that fetal movements are present, and denies leakage of fluid or vaginal bleeding. She has not done any prior prenatal visit. She has Positive Human Immunodeficiency virus test done in the labour and delivery room (DGH). Patient admitted with a vaginal exam (VE) showing Cervix dilated at 1 cm, 40% effaced and station −5.

Past obstetric history: negative.

#### Past gynecological history

Menarche at 11 years old, menstrual is cycle 25 ± 4 days, regular. Had never done a Pap smear nor taken contraception. She is blood group B Rhesus positive, declares no allergies and received no anti-malaria prophylaxis or iron supplements during this pregnancy.

Social history: Denies history of alcohol, smoking, drug abuse and has had one sexual partner.

Family history: Parents are married and do subsistence farming. Declares no history of hypertension or diabetes mellitus.

Review of system: uterine contractions.

#### Physical examination

- General appearance: alert and oriented.
- Vital signs: Temperature 37.6 °C, BP 120/70 mmHg, Pulse 72 bpm.
- HEENT: No scleral icterus, pale conjunctiva.
- Neck: normal; lungs: normal; heart: normal; breasts: symmetric, no masses.
- Abdomen: gravid, non-tender.
- Presentation: vertex.
- Extremities: no lower extremity edema.
- Pelvis: adequate.
- Sterile speculum exam (SSE): membranes intact, no vaginal discharge.
- Sterile vaginal exam (SVE): cervix 1 cm dilated, 40% effaced, station −5.
- Ultrasound: vertex presentation confirmed, posterior placenta, no fetal anomalies.
- Fetal monitor: not done.

#### Assessment

20½-year old G1P0 at 33 weeks GA presented with uterine contractions q 10 min.

1. Preterm labor in latent phase.
2. HIV positive.

#### Plan

1. Admit to labor and delivery room.
2. Nothing per os.
3. D5 RL 125 ml/h.
4. Lab: Full blood count (FBC), thick and thin films for malaria, blood electrolytes, coagulation studies, CD4 count, Viral load (VL), aspatate aminotransferase, alanine aminotransferase, blood urea nitrogen (BUN),
5. Antiretroviral therapy.
6. Type and cross-match blood.
7. Prepare for cesarean delivery.

#### Results

 Hemoglobin: 6.5 g/dl, hematocrit: 19.3%, platelet count: 75,000, CD4: 195, VL 18,000 copies/ml. CD4 and VL results were received after the cesarean delivery.

She underwent a cesarean delivery of a live-born male. The 5 min Apgar score was 7 and estimated blood loss was 650 ml. She was transfused 1500 ml packed cells. Hospital stay was 6 days. Mother received bromocriptine for breast milk suppression and baby received exclusive formula milk. HIV testing at 18 months was negative.

### Case 6

MA, a 27-year-old primigravida at 36 weeks’ gestation dated by last menstrual period admitted to our Department with uterine contractions q 15 min. She has no formal education but is a trader and single. She reports that fetal movements are present, and denies leakage of fluid or vaginal bleeding. She had one prior prenatal visit where she was diagnosed positive for the Human Immunodeficiency virus. Patient admitted with a vaginal exam (VE) showing cervix dilated at 1 cm, 50% effaced and station −5.

Past obstetric history: negative.

#### Past gynecological history

Menarche at 13 years old, menstrual is cycle 30 days, regular. Had never done a Pap smear nor taken contraception. She is blood group A Rhesus positive, declares no allergies and received no anti-malaria prophylaxis or iron supplements during this pregnancy.

Social history: Denies history of alcohol, smoking, drug abuse and has had six sexual partners.

Family history: Parents are married and do business. Declares no history of hypertension or diabetes mellitus.

Review of system: uterine contractions.

#### Physical examination

- General appearance: alert and oriented.
- Vital signs: Temperature = 37.6 °C, BP 120/70 mmHg, Pulse 74 bpm.
- HEENT: No scleral icterus, pale conjunctiva.
- Neck: normal; lungs: normal; heart: normal; breasts: symmetric, no masses.
- Abdomen: gravid, non-tender.
- Presentation: vertex.
- Extremities: no lower extremity edema.
- Pelvis: adequate.
- Sterile speculum exam (SSE): membranes intact, no vaginal discharge.
- Sterile vaginal exam (SVE): cervix 1 cm dilated, 50% effaced, station −5.
- Ultrasound: vertex presentation confirmed, posterior placenta, no fetal anomalies.
- Fetal monitor: not done.

#### Assessment

27-year old G1P0 at 36 weeks GA presented with uterine contractions q 15 min.

1. Preterm labor in latent phase.
2. HIV positive.

#### Plan

1. Admit to labor and delivery room.
2. Nothing per os.
3. D5 RL 125 ml/h.
4. Lab: Full blood count (FBC), thick and thin films for malaria, blood electrolytes, coagulation studies, CD4 count, Viral load (VL), aspatate aminotransferase, alanine aminotransferase, blood urea nitrogen (BUN),
5. Antiretroviral therapy.
6. Type and cross-match blood.
7. Prepare for cesarean delivery.

#### Results

Hemoglobin: 8.5 g/dl, hematocrit: 26%, platelet count: 100,000, CD4: 200, VL 85,400 copies/ml.

She underwent a cesarean delivery of a live-born male. The 5 min Apgar score was 9 and estimated blood loss was 750 ml and birthweight 2100 g. Hospital stay was 6 days. Mother received bromocriptine for breast milk suppression, hematinics and baby received exclusive formula milk. The baby was HIV negative at 18 months.

### Case 7

EE, a 33-year-old primigravida at 38 weeks’ gestation dated by last menstrual period admitted to our Department with generalized body weakness, fever and uterine contractions q 10 min. She is married and was diagnosed HIV positive at the beginning of the current pregnancy and since then refused going to hospital. She reports that fetal movements are present, and denies leakage of fluid or vaginal bleeding. Patient admitted with a vaginal exam (VE) showing Cervix closed, long and station −5.

Past obstetric history: negative.

#### Past gynecological history

Menarche at 12 years old, menstrual is cycle 29 days, regular. Had never done a Pap smear nor taken contraception. She is blood group AB Rhesus positive, declares no allergies and received no anti-malaria prophylaxis or iron supplements during this pregnancy.

Social history: Denies history of alcohol, smoking, drug abuse and has had one sexual partner.

Family history: Parents are married. Declares no history of hypertension or diabetes mellitus.

Review of system: Uterine contractions, fever and body weakness.

#### Physical examination

- General appearance: alert and oriented.
- Vital signs: Temperature = 39 °C, BP 130/80 mmHg, Pulse 90 bpm.
- HEENT: No scleral icterus, pale conjunctiva.
- Neck: normal; lungs: normal; heart: normal, breasts: symmetric, no masses.
- Abdomen: gravid, non-tender.
- Presentation: vertex.
- Extremities: no lower extremity edema.
- Pelvis: adequate.
- Sterile speculum exam (SSE): membranes intact, no vaginal discharge.
- Sterile vaginal exam (SVE): cervix closed, long, station −5.
- Ultrasound: vertex presentation confirmed, posterior placenta, no fetal anomalies.
- Fetal monitor: not done.

#### Assessment

A 33-year old G1P0 at 38 weeks GA presented with uterine contractions q 10 min.

1. Preterm labor in latent phase.
2. Pyrexia of unknown origin.
3. HIV positive.

#### Plan

1. Admit to labor and delivery room.
2. Nothing per os.
3. D5 RL 125 ml/h.
4. Lab: Full blood count (FBC), thick and thin films for malaria, blood electrolytes, coagulation studies, CD4 count, Viral load (VL), Aspatate aminotransferase, alanine aminotransferase, blood urea nitrogen (BUN), Widal serology, urinalysis and culture, vaginal smear and cultue.
5. Antiretroviral therapy.
6. Type and cross-match blood.
7. Prepare for cesarean delivery.

#### Results

Hemoglobin: 7.9 g/dl, hematocrit: 23.5%, platelet count: 130,000, CD4: 254, VL 9400 copies/ml, BUN: 0. 23 g/l, creatinine 11.5 mg/dl, AST: 25 IU/l, ALT: 27 IU/l. Positive for malaria parasite (*Plasmodium falciparum*).

Quinine infusions were begun and she underwent a cesarean delivery of a live-born female. The 5 min Apgar score was 10 and estimated blood loss was 450 ml and birth weight 1980 g. Hospital stay was 5 days. Mother received bromocriptine for breast milk suppression, iron and folic acid and baby received exclusive formula milk. HIV testing of the baby at 18 months was negative.

### Cesarean section technique

**Fig. 1:**
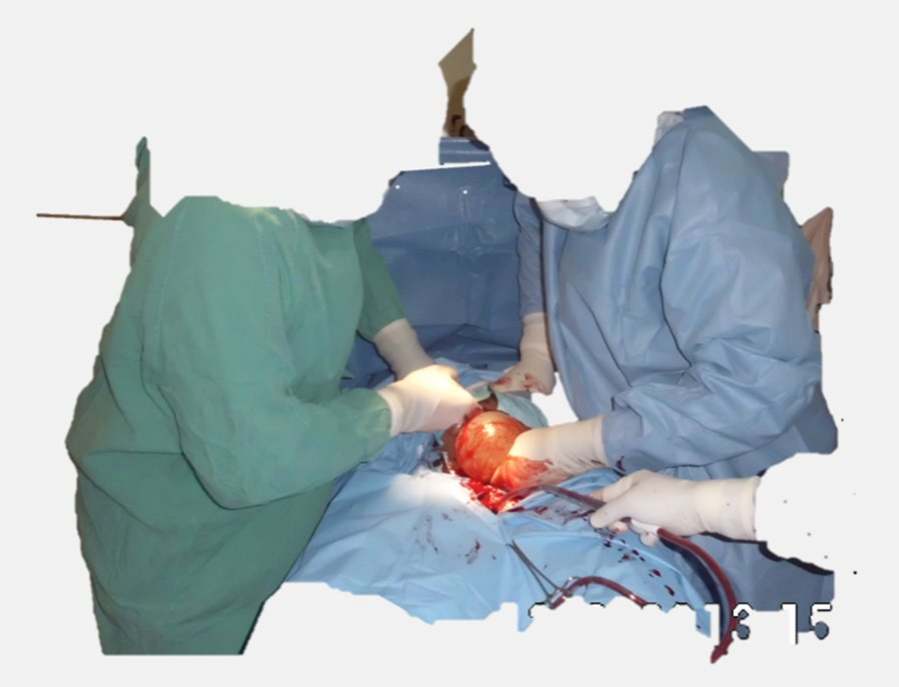
Delivery of fetal head without rupture of amniotic membranes

**Fig. 2:**
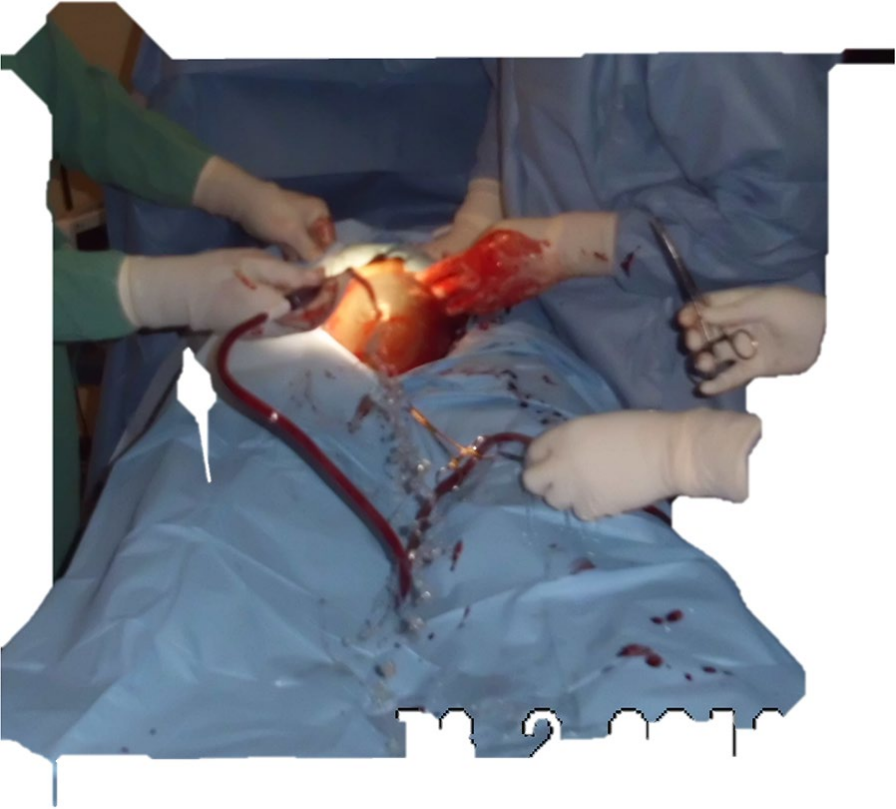
Rupture of amniotic membranes after delivery of fetal head

**Fig. 3:**
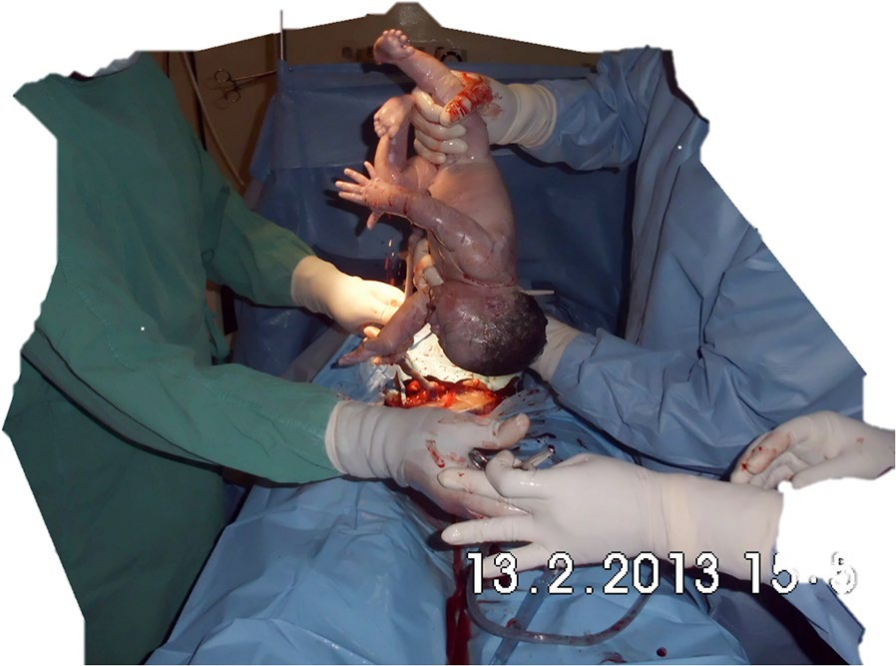
Delivery of baby

All patients were operated as emergencies under regional anesthesia (epidural ± spinal anesthesia) using bupivacaine hydrochloride (Macaine^®^0.5%). A low transverse skin incision was used in all the cases with cauterization for hemostasis and blunt dissection for peritoneal entry. The vesico-uterine peritoneal fold was dissected and a Doyen retractor placed between the uterus and bladder to protect the bladder from injury. A transverse lower segment uterine incision was made gently until the endometrium was seen bulging. The endometrium was entered and enlarged by blunt dissection without rupture of amniotic membranes. During the cesarean section the fetal head was delivered through the uterine incision without rupture of amniotic membranes (Fig. 1). The amniotic membranes were ruptured after delivery of the fetal head (Fig. 2), and then the rest of the body was delivered (Fig. 3).

The umbilical cord was clamped and sectioned without milking of cord blood towards the fetus. The baby was then handed to the pediatrician without suctioning. Antibiotic prophylaxis (ceftriaxone 2 gm) was administered to the mother after sectioning of the umbilical cord. The placenta was delivered by controlled cord traction and uterine massage. The uterine cavity was cleansed with gauze mounted on ring forceps; then intra-abdominal uterine repair was done by single layer closure with Polyglactin-910 (Vicryl No. 1). Peritoneal toileting was done with warm saline and closure of the abdominal fascia was done with Polyglactin-910 (Vicryl No. 2). The subcutaneous layer was not closed and the skin was approximated with continuous sub cuticle absorbable sutures (Vicryl rapide^®^ 2-0).

## Discussion

The aim of this case series was to show that HIV positive women with sub-optimal ANC uptake could benefit from elective cesarean section by reducing MTCT of HIV. The average age of our patients was 21.93; range 20.5–33 years. Most of the women 5/7 (71.4%) were aged between 20.5 and 26 years. The mean gestation age was 34.71; range 33–38 weeks; mean birth weight 2080; range 1980–2350 g. The mean hemoglobin concentration was 6.54; range 5.0–8.5 g/l; mean hematocrit 19.64%; range 14.9–26%; mean platelet count 89,290; range 65,000–130,000. Similarly, the mean CD4 count 190.57; range 143–254; mean VL 26,114.3; range 9400–85–400 copies/ml. Furthermore, the mean blood loss 650; range 450–800 ml; mean blood transfused 1285.7; range 0–2000 ml; mean 5 min Apgar score 7; range 5–10; mean hospital stay 7; range 5–12 days. Four, 57.14% patients were diagnosed and treated for malaria.

### Socio-demographic characteristics of participants

The average age of study participants was 21.93 years. Studies in certain rural areas of South Africa have found that young pregnant women are HIV infected at surprisingly high rates: 18.6% of 17- to 18-year-olds, 25.4% of 19–20-year-olds, 32.8% of 21- to 22 year-olds, and 44.8% of 23- to 24-year-olds [17]. Adolescent girls and young women face a lethal mix of legal, economic, and social factors known as structural drivers that interact to affect behavior and decisions on sexual partners. These factors include gender inequality and gender roles, lack of livelihood options, and stigma and discrimination, which reduce the benefits conferred by enabling factors such as education, economic assets, and legal protection. Structural drivers also lead to early, coerced, and intergenerational sex, transactional sex, child marriage, and gender-based violence and exploitation. The drivers also interfere with girls’ and young women’s ability to use and adhere to biomedical prevention technologies [17]. Most participants were not married, unemployed, and for the most part were uneducated or had primary education. This conforms with other studies [18] Previous studies have reported very high prevalence of HIV amongst this group [19]. There is therefore need for expanded PMTCT programmes in Cameroon.

### Obstetric characteristics of participants

The mean gestational age at admission and delivery was 34.71 weeks. Several studies have reported the association between HIV infection and preterm delivery [20].The problem is also confounded by the presence of associated malaria infection among (4/7) 57.14% cases. Cameroon is a hyperendemic zone for malaria transmission. The 2014 WHO statistics for Cameroon report a high malaria transmission 71% (>1 case per 1000 population) [21]. Other authors in Kenya reported that malaria was associated with both intrauterine growth restriction (IUGR) and preterm delivery (PTD), resulting in a reduction in mean birthweight of 145 g (95% CI 82–209) and 206 g (95% CI 115–298), respectively among HIV-seronegative pregnant women and the dual association was associated with poor obstetric outcomes including anemia [22].

Case 1 was diagnosed to have bacterial vaginosis (BV), a known risk factor for preterm deliveries and preterm premature rupture of fetal membranes [23–25]. The mean hemoglobin concentration and hematocrit was 6.54 g/dl and 19.64%, respectively. This could explain the increased use of blood transfusion; average blood transfused was 1285.7 ml although the average blood loss was 650 ml. The average 5 min Apgar score of the cases was low = 7 and the average birthweight of the babies was 2080 g. This was small for an average gestational age of 34.7 weeks. This shows that some of these babies were small for date or had intrauterine growth restriction because of chronic fetal distress. This could be exemplified by the case that gave birth at 38 weeks gestation to a 1980 g baby but with Apgar score 10 and was one of the cases with malaria co-infection.

The average viral load was 26,114.3 copies/ml and mean CD4 count 190.57 cells/mm^3^. This was not particularly high as expected for patients who had not been on ARV treatment prior to admission. It is worth noting that the results of these two examinations came after the cesarean sections had been done in all the cases.

A protective effect of elective cesarean delivery had been proven in the absence of antiretroviral (ARV) therapy or with zidovudine monotherapy, leading to 2- to 5-fold reduction in mother-to-child transmission (MTCT) of human immunodeficiency virus (HIV) compared to vaginal delivery, both in large observational studies and in randomized clinical trials [26, 27]. Elective CS at 38 gestational weeks was thus recommended for all HIV-infected women in all industrialized countries from 1997 through 1998 [13, 28] although this is no longer the case for women receiving highly active antiretroviral therapy (HAART) [27].

In the DGH, all women who come to give birth undergo a pretest counseling and subsequent HIV testing using the Determine test strips including post-test counseling. The recommended mode of delivery for women receiving HAART is vaginal. In a previous retrospective study carried out at the DGH during the period 2002–2007, a total of 5261 deliveries were studied and 138 (2.62%) were from HIV positive mothers. Of the 138 babies, only 87 (63%) were tested for HIV and 4 (4.6%) tested HIV positive at 18 months [12]. Among these four cases, only one had appropriate PMTCT follow-up during pregnancy. This gave us a transmission rate of 1.19% after PMTCT follow-up in pregnancy [12].

Several observational studies and meta-analyses have suggested that there is no longer any additional benefit from elective CS beyond that conferred by combination ART and viral suppression [26, 29]. Since then, most low-income countries have adopted the policy of vaginal delivery. But there are still some patients who either do not subscribe to or are noncompliant with antiretroviral treatment. Women who delivered in our department were explained the available options regarding feeding of their babies. Those who could afford formula feeding were encouraged to do exclusive formula feeding. This practice conforms with the recommendations of other studies [30, 31]. However, in Cameroon the Ministry of Health recommends exclusive breastfeeding. Several cesarean delivery techniques have been proposed, some of which have proven short-term advantages over the classical Pfannenstiel procedure with regard to operation time, bleeding and morbidity [2]. All our patients were operated as emergencies using a low transverse skin incision. We believe that the crucial moment when performing CS for PMTCT of HIV is during delivery of the fetal head. We used regional anesthesia in all the women to allow the surgeon to operate slowly and avoid the babies inhaling the mother’s blood. Other studies have shown that in the absence of risk factors like rupture of membranes, the risk of MTCT of HIV is negligible even in patients who are not receiving combination ARV therapy. Proof of this is that all babies born by CS in our series were HIV negative at 18 months. We did not have any accidental rupture of the fetal membranes during cesarean section prior to delivery of the fetal head in our series.

The limitation to this case series is that we did not study the duration of stay of newborn babies in the neonatal intensive care unit after cesarean delivery. The CD4 and VL results were all obtained after the surgical procedure. These values were particularly low as compared to patients who were not under ARV treatment. This may raise the question of the test procedure and equipment.

## Conclusions

Most of the study participants had multiple risk factors for preterm labour. When a good cesarean section technique is used in women with high viral load and low CD4 counts, risk of MTCT HIV are greatly reduced even in low-income countries. We recommend that multicentre studies be carried out to validate this technique for wider use. CARE guidelines/methodology were adhered to in the preparation of the manuscript.

## Abbreviations

CS: cesarean section
PMTCT: prevention of mother-to-child transmission
HIV: human immunodeficiency virus
VL: viral load
DGH: Douala General Hospital
